# Gut Microbiota and Colorectal Cancer: An Umbrella Review of Methodological Trends and Clinical Correlations

**DOI:** 10.7759/cureus.54210

**Published:** 2024-02-14

**Authors:** Alousious Kasagga, Chnoor Hawrami, Erica Ricci, Kirubel T Hailu, Korlos Salib, Sanath Savithri Nandeesha, Pousette Hamid

**Affiliations:** 1 Pathology, California Institute of Behavioral Neurosciences & Psychology, Fairfield, USA; 2 Pediatric Surgery, California Institute of Behavioral Neurosciences & Psychology, Fairfield, USA; 3 Anesthesiology, California Institute of Behavioral Neurosciences & Psychology, Fairfield, USA; 4 Internal medicine, California Institute of Behavioral Neurosciences & Psychology, Fairfield, USA; 5 Internal Medicine, Afet Speciality Clinic, Addis Ababa, ETH; 6 Internal Medicine, St Mary El zaytoun, Cairo, EGY; 7 Internal Medicine, Karnataka Institute of Medical Sciences, Hubli, IND; 8 Internal Medicine, California Institute of Behavioral Neurosciences & Psychology, Fairfield, USA; 9 Neurology, California Institute of Behavioral Neurosciences & Psychology, Fairfield, USA

**Keywords:** fusobacterium nucelatum, colorectal neoplasm, microbiota dysbiosis, gut microbiome, colorectal cancer

## Abstract

In this umbrella review, we analyze the effect of gut microbiota on the development and progression of colorectal cancer (CRC), a global health challenge. Following Preferred Reporting Items for Systematic Reviews and Meta-Analysis (PRISMA) 2020 guidelines, we searched multiple databases for the most relevant systematic reviews and meta-analyses from 2000 to 2023. We identified 20 articles that met our inclusion criteria. The findings include the identification of specific microbiota markers, such as *Fusobacterium nucleatum*, for potential early diagnosis and improvement of disease treatment. This thorough study not only establishes the connection between microbiota and CRC but also provides valuable knowledge for future research in developing microbiome-centered treatments and preventive methods.

## Introduction and background

Colorectal cancer (CRC) ranks as the second cause of cancer mortality in the United States and the third worldwide. Statistics indicate that in 2023, the estimated number of new cases will be 153,020, with 52,550 deaths [1]. Alarmingly, by 2040, the global incidence of CRC will skyrocket to 3.2 million new cases, with 1.6 million deaths [2]. The development of CRC is a multifaceted process involving genetics, lifestyle, age, and environmental factors. Interestingly, 85-90% of the CRC cases are related to environmental factors rather than genetics [3]. The gut microbiota especially plays a critical role that spans from the initiation to the evolution of the disease [4,5].

Recent studies have highlighted the differences in the gut microbiome in healthy individuals compared to those with the disease, ranging from essential identification of the gut microbiome to utilizing and manipulating treatments such as probiotics, prebiotics, and fecal transplants. Even though these techniques show potential, assessing their risks and patient-specific aspects is crucial [6,7]. Despite the advancements, we need a more profound understanding of which microbiota and by which mechanism leads to -to advance better-targeted therapies [8].

This umbrella review combines and evaluates previous studies to understand how different types of gut microbiota composition influence CRC development and advancement. We will investigate the potential of early diagnosis and individualized treatments using microbiota profiling. We aim to shed light on future directions to enhance public health strategies in CRC control by bridging the gaps between current research and clinical applications.

## Review

Methodology

We conducted this umbrella review strictly adhering to the Preferred Reporting Items for Systematic Review and Meta-Analysis (PRISMA) 2020 guidelines [9].

Search Strategy

To identify relevant systematic reviews, we searched the following electronic databases: Google Scholar, PubMed, Web of Science, and Science Direct. We focused on two key topics: microbiota and colorectal cancer. Our search strategy included Medical Subject Heading (MeSH) terms and relevant keywords. Table 1 presents the selected keywords and MeSH terms related to both topics.

**Table 1:** Selected keywords and MeSH terms for microbiota and colorectal cancer MeSH: Medical Subject Heading

	Microbiota	Colorectal cancer
Keywords	Microbiota, Microbiome, Gut flora, Intestinal Bacteria, Intestinal Microbiome, Gut Bacteria, Dysbiosis	Colorectal Cancer, Colorectal Neoplasms, Colorectal Carcinoma, Colon Cancer, Rectal Cancer, Colorectal Tumor
MeSH terms	Microbiota [MeSH], Gastrointestinal Microbiome [MeSH], Dysbiosis [MeSH]	Colorectal Neoplasms [MeSH], Neoplastic Processes [MeSH], Colonic Neoplasms [MeSH], Rectal Neoplasms [MeSH]

A search query was created using the identified keywords and MeSH terms, using advanced search and Boolean operators (AND, OR). (Microbiota OR Microbiome OR Gut flora OR Intestinal bacteria OR Intestinal microbiome OR Gut bacteria OR Dysbiosis Microbiota [MeSH] OR Gastrointestinal Microbiome [MeSH] OR Dysbiosis [MeSH]) AND (Colorectal cancer OR Colorectal neoplasms OR Colorectal carcinoma OR Colon cancer OR Rectal cancer OR Colorectal tumor OR Colorectal Neoplasms [MeSH] OR Neoplastic Processes [MeSH] OR Colonic Neoplasms [MeSH] OR Rectal Neoplasms [MeSH])

Inclusion and Exclusion Criteria

Inclusion criteria included systematic reviews and meta-analyses that examine the relationship between gut microbiota alterations and CRC risk and progression in adult populations aged 18 years and older. We only considered studies that have been published in peer-reviewed journals and are in the English language. Exclusion criteria included narrative reviews, editorials, opinion pieces, case reports, studies in pediatric populations, or studies that did not distinguish CRC from other cancer types. We completed the comprehensive search after manually reviewing the reference lists of included articles published from January 1, 2000, to December 1, 2023.

Data Extraction

Our umbrella review used a Microsoft Excel worksheet (Microsoft Corporation, Redmond, Washington) for data extraction and analysis. Two reviewers (Alousious Kasagga and Chnoor Hawrami) independently screened titles and abstracts to determine their eligibility. We then conducted a full-text review to confirm their inclusion. The extracted data included the author(s), year of publication, journal name, funding source, references to the included studies, population characteristics, method of microbiota assessment, measured CRC outcomes, key findings, and conclusions. If there were any inconsistencies, they were resolved through consultation with a third reviewer (Erica Ricci) when deemed necessary.

Quality Assessment

We used the AMSTAR 2 (A Measurement Tool to Assess Systematic Reviews) tool to evaluate the quality of the included systematic reviews [10].

Evaluation of Study Overlap in Systematic Reviews

We used a comprehensive approach to measure the degree of study duplication among the systematic studies. This approach involved three primary metrics: overlap percentage (%), covered area (CA), and corrected covered area (CCA) [11]. Overlap percentage (%) measures the proportion of primary studies cited in several systematic reviews. It is calculated by dividing the number of repeated primary studies by the total number of primary studies. It provides a clear indicator of the level of study overlap. The CA measures the scope of the research field covered by the included reviews. It is calculated by dividing the total number of citations by the product of the total number of primary studies and the number of included reviews. CCA refines the CA by considering the frequency of each primary publication across the included reviews. Assessing the degree of overlap using this metric offers a greater level of accuracy, classifying it as low (0-5%), moderate (6-10%), high (11-15%), and very high (>15%).

$Overlap\:percentage\:(\%)=\frac{the\:number\:of\:repeated\:primary\:studies}{total\:number\:of\:primary\:studies}$

$CA=\frac{total\:number\:of\:citations}{(total\:number\:of\:primary\:studies)\times (total\:number\:of\:included\:reviews)}$

$CCA=\frac{(total\:number\:of\:citations)-(total\:number\:of\:primary\:studies)}{(total\:number\:of\:primary\:studies)\times (total\:number\:of\:included\:reviews\:-\:1)}$

*Ethical Considerations* Since an umbrella review does not involve primary data collection but instead synthesizes data from previously published systematic reviews, there is no need for ethical approval.

Results

Search Results

**Figure 1:**
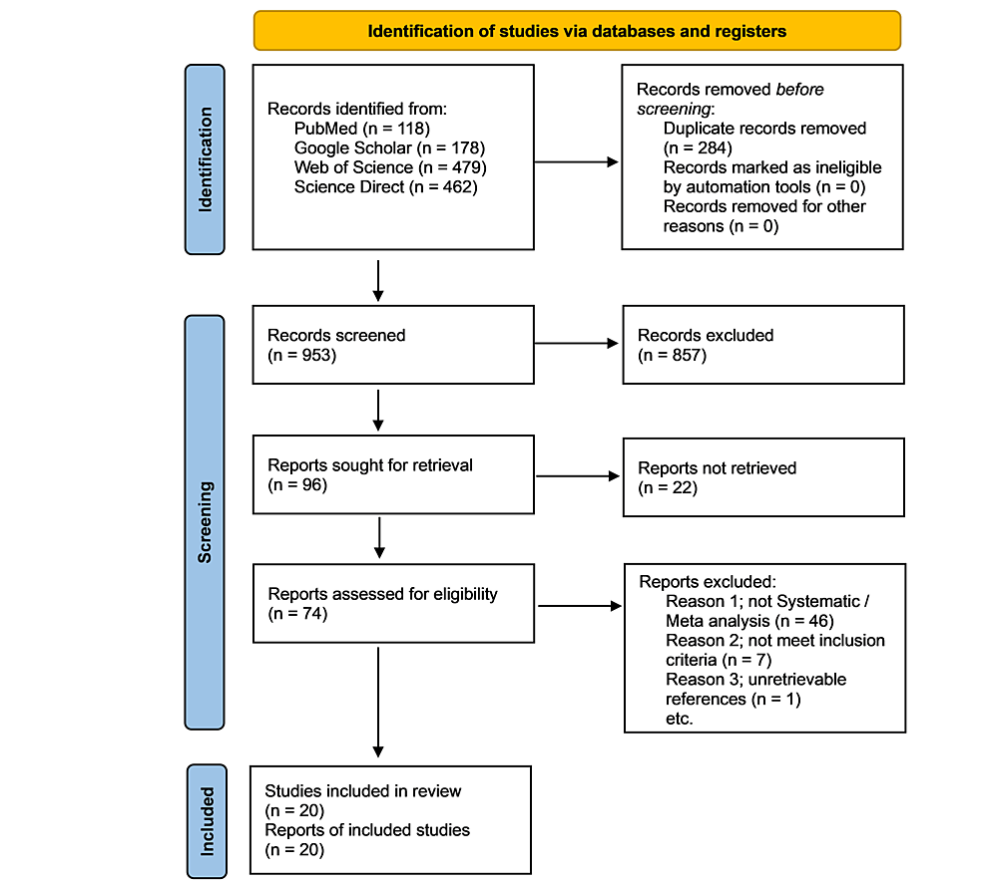
PRISMA flow diagram. PRISMA: Preferred Reporting Item for Systematic Reviews and Meta-Analyses The diagram was drawn by the authors of this article.

The initial search yielded a total of 1,237 records. After removing the duplicates, 953 records were screened based on their titles and abstracts. Out of the 96 reports that were selected for full-text review, 22 of them were not obtainable. We assessed the eligibility of all 74 articles and identified only 20 systematic reviews and meta-analyses that met our inclusion criteria for the final synthesis [12-31]. Figure 1 represents the PRISMA flow diagram with a detailed search and selection process overview.

Study Characteristics

The included reviews covered a range of topics, exploring specific strains of gut microbiota, overall microbial diversity, and their impact on CRC risk and progression. Table 2 summarizes each selected study's characteristics, containing the author's name, journal name, publication year, number of primary studies included, funding source, study objective, and outcome.

**Table 2:** Characteristics of included reviews CRC: colorectal cancer

Author	Journal and Year	Studies	Funding Source	Objective of Study	Outcome
Alhhazmi et al. [12]	Microorganisms, 2023	24	Ministry of Education in Saudi Arabia	Finding specific gut microbial markers and related metabolites that could serve as diagnostic indicators for CRC	They identified nine distinct microbial markers in CRC patients compared to healthy controls.
Amitay et al. [13]	Gut Microbes, 2018	19	No funding	Analyzing the association between the gut microbiota in fecal samples and colorectal neoplasms detection	Variations in fecal gut microbiota can be used in early noninvasive CRC detection.
Anandakumar et al. [14]	Updates in Surgery, 2019	4	No funding	Examining the role of the fungal microbiome in CRC	Specific fungal microbiomes, such as Ascomycota and Basidiomycota phyla, can be used as fungal biomarkers for CRC detection.
Aprile et al. [15]	Cancers (Basel), 2021	19	No funding	Examining changes in microbiota in precancerous colon lesions and their potential role in CRC development	They found a high abundance of Proteobacteria and Fusobacteria in precancerous CRC lesion development.
Borges-Canha et al. [16]	Revista Espanola De Enfermedades Digestivas 2015	31	Not mentioned	Examining the function of colonic microbiota in CRC development	They found that some bacteria, such as Fusobacteria and Alistipes, increased while others, such as Bifidobacterium and Lactobacillus, decreased during CRC development.
Costa et al. [17]	Cancers (Basel), 2022	39	Fundação para a Ciência e a Tecnologia	Focusing on the role of tissue-associated microbiota in CRC	They identified 12 microbial taxa positively and 18 taxa negatively associated with CRC.
Eastmond et al. [18]	Cureus, 2022	13	No funding	Examining the role of the gastrointestinal microbiome in CRC	Microbiome alterations in the gastrointestinal tract (including the oral cavity) lead to CRC development.
Fratila et al. [19]	Medicine and Pharmacy Reports, 2023	50	Not mentioned	Evaluating the role of probiotics in microbiota modulation and its effect on CRC control	Probiotics and prebiotics, such as Lactobacilli and Bifidobacteria, show potential in CRC control by modulating microbiota.
Gethings-Behncke et al. [20]	Cancer Epidemiology, Biomarkers & Prevention, 2020	45	Not mentioned	Examining the correlation between *Fusobacterium nucleatum* in the colorectum and its risk of developing CRC	They found abundant *F. nucleatum* in CRC tissue and fecal samples. Also, those with elevated levels of this bacterium had a poorer prognosis.
Hussan et al. [21]	World Journal of Gastroenterology, 2017	90	Not mentioned	Examining the relationship between Fusobacterium and colorectal malignancy	They suggest that a specific strain of Fusobacterium plays an active role in CRC.
Karimi et al. [22]	Iranian Journal of Colorectal Research, 2020	54	Not mentioned	Examining changes in microbiota composition in the feces and mucosa of individuals with CRC	Their result shows that some of the taxa they examined grew more in CRC patients than in healthy controls. Conversely, some of the taxa were less seen.
Mohammad et al. [23]	Medical Journal of Malaysia, 2023	7	Not mentioned	Reviewing the CRC risk associated with *Streptococcus gallolyticus*	They concluded there is insufficient evidence for *S. gallolyticus* as a CRC risk factor.
Negrut et al. [24]	Microorganisms, 2023	14	University of Oradea	Investigating the potential of using oral microbiome indicators to diagnose and predict the course of CRC	They suggested the use of salivary *F. nucleatum* DNA for noninvasive CRC diagnosis.
Peng et al. [25]	Chinese Medical Journal (Engl), 2018	7	No funding	Examining the diagnostic accuracy of intestinal *F. nucleatum* in CRC	They proved that intestinal *F. nucleatum* is a key marker for CRC diagnosis.
Ranjbar et al. [26]	Cancer Cell International, 2021	39	Isfahan University of Medical Science	Examining the dysbiosis signature of *F. nucleatum* in CRC, and analyzing its consequences	Their data suggested that *F. nucleatum* is a prognostic biomarker and potential target for antibiotic treatment.
Tabowei et al. [27]	Cureus, 2022	9	No funding	Evaluating if an asymmetry in the microbiota contributes to the development of CRC	They concluded Fusobacterium increases in CRC patients and reduces in healthy individuals.
Valciukiene et al. [28]	Cancers (Basel), 2023	32	Research Council of Lithuania	Analyzing and contrasting the dysbiosis of bacteria collected from tissue samples vs fecal samples in individuals with precancerous colorectal lesions	They found a high correlation of gut microbiota changes in tissue and fecal samples with an abundant of Fusobacterium.
Von Vorstenbosch et al. [29]	Metabolites, 2022	76	European Commission and Pentax Medical	Examining the gut microbiota's role in producing volatile metabolic compounds associated with colorectal neoplasia	They found that the gut microbiota affects the volatile organic compounds in CRC patients.
Yu et al. [30]	British Journal of Cancer, 2022	75	Cancer Research UK and National Natural Science Foundation of China	Identifying microbial markers for risk prediction of colorectal neoplasia	Nine fecal and two oral microbiotas, as well as serum antibodies, were associated with CRC diagnosis.
Zwezerijnen-Jiwa et al. [31]	Neoplasia, 2023	28	No funding	Identifying microbiome markers for the early diagnosis of CRC	Combination of traditional early detection tests (such as the guaiac fecal occult blood test) and microbiome markers from stool samples performed better for CRC detection.

Quality Evaluation of the Included Reviews

We used the AMSTAR 2 assessment tool to evaluate the reviews' quality. Three reviews were of high quality, while 17 were of moderate quality. The most common observed limitation in the moderate reviews was not performing data searches and extraction in duplicate, not registering their protocols, and failing to report. Table 3 shows how each study performed during the AMSTAR 2 evaluation.

**Table 3:** Quality evaluation using AMSTAR 2 checklist questions Y: Yes; N: No; PY: Partial Yes; NA: Not applicable; AMSTAR: A Measurement Tool to Assess Systematic Reviews

STUDY	AMSTAR 2 Questions															
	1	2	3	4	5	6	7	8	9	10	11	12	13	14	15	16
Alhhazmi et al. [12]	Y	PY	Y	Y	Y	Y	Y	Y	Y	Y	NA	NA	Y	Y	Y	Y
Amitay et al. [13]	Y	PY	Y	Y	Y	Y	Y	Y	Y	Y	NA	NA	Y	Y	Y	Y
Anandakumar et al. [14]	Y	PY	Y	Y	N	N	Y	Y	Y	Y	NA	NA	Y	Y	Y	Y
Aprile et al. [15]	Y	PY	Y	Y	Y	Y	Y	Y	Y	Y	NA	NA	Y	Y	Y	Y
Borges-Canha et al. [16]	Y	PY	Y	Y	N	N	Y	Y	Y	Y	NA	NA	Y	Y	Y	N
Costa et al. [17]	Y	PY	Y	Y	Y	Y	Y	Y	Y	Y	NA	NA	Y	Y	Y	Y
Eastmond et al. [18]	Y	PY	Y	Y	Y	Y	Y	Y	Y	Y	NA	NA	Y	Y	Y	Y
Fratila et al. [19]	Y	PY	Y	Y	Y	Y	Y	Y	Y	Y	NA	NA	Y	Y	Y	N
Gethings-Behncke et al. [20]	Y	PY	Y	Y	Y	Y	Y	Y	Y	Y	Y	Y	Y	Y	Y	Y
Hussan et al. [21]	Y	PY	Y	Y	Y	Y	Y	Y	Y	Y	NA	NA	Y	Y	Y	Y
Karimi et al. [22]	Y	PY	Y	Y	Y	Y	Y	Y	Y	Y	NA	NA	Y	Y	Y	Y
Mohammad et al. [23]	Y	PY	Y	Y	Y	Y	Y	Y	Y	Y	NA	NA	Y	Y	Y	N
Negrut et al. [24]	Y	Y	Y	Y	Y	Y	Y	Y	Y	Y	NA	NA	Y	Y	Y	Y
Peng et al. [25]	Y	PY	Y	Y	Y	Y	Y	Y	Y	Y	Y	Y	Y	Y	Y	Y
Ranjbar et al. [26]	Y	PY	Y	Y	N	N	Y	Y	Y	Y	NA	NA	Y	Y	Y	Y
Tabowei et al. [27]	Y	PY	Y	Y	Y	Y	Y	Y	Y	Y	NA	NA	Y	Y	Y	Y
Valciukiene et al. [28]	Y	Y	Y	Y	Y	Y	Y	Y	Y	Y	NA	NA	Y	Y	Y	Y
Vorstenbosch et al. [29]	Y	PY	Y	Y	Y	Y	Y	Y	Y	Y	NA	NA	Y	Y	Y	Y
Yu et al. [30]	Y	Y	Y	Y	Y	Y	Y	Y	Y	Y	NA	NA	Y	Y	Y	Y
Zwezerijnen-Jiwa et al. [31]	Y	PY	Y	Y	Y	Y	Y	Y	Y	Y	NA	NA	Y	Y	Y	Y

Analysis of Study Overlap in Included Reviews

We reviewed a total of 662 citations, comprising 414 distinct primary studies, and identified 113 instances where primary studies were repeated in our 20 systematic reviews and meta-analyses. The most commonly cited study appeared in eight reviews. The calculation of the overlap parameters is as follows.

$Overlap\:percentage (\%)=\frac{113}{414}=27.29\%$

$Covered\:area\:(CA)=\frac{662}{414\times 20}=7.99\%$

$Corrected\:covered\:area\:(CCA)=\frac{662-414}{414\times (20-1)}=3.15\%$

The overlap percentage of 27.29% indicates a significant redundancy, which means that more than a quarter of the primary publications are repetitive. The 7.99% CA shows a low redundancy, indicating that our included reviews are unique regarding their content, data, or findings, providing diverse and complete literature coverage. CCA is a more accurate measure for overlap analysis, with 3.15% indicating a low degree of overlap.

**Figure 2:**
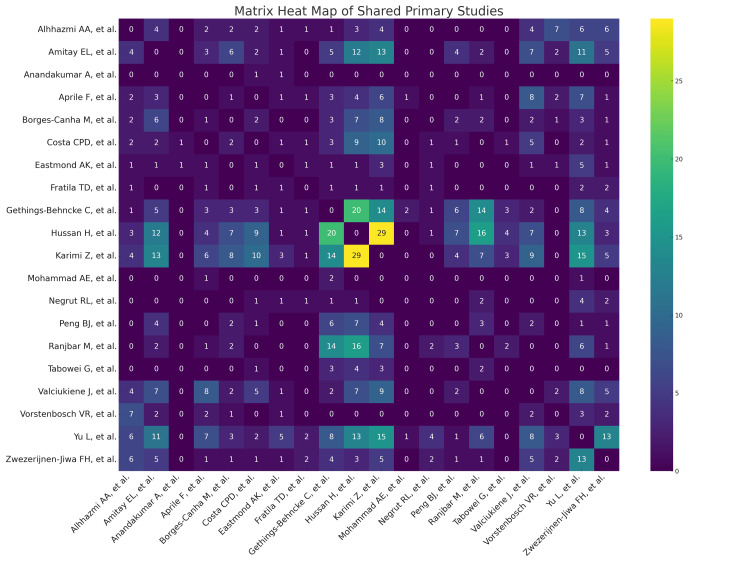
Matrix heatmap of included reviews Alhhazmi et al. [12]; Amitay et al. [13]; Anandakumar et al. [14]; Aprile et al. [15]; Borges-Canha et al. [16]; Costa et al. [17]; Eastmond et al. [18]; Fratila et al. [19]; Gethings-Behncke et al. [20]; Hussan et al. [21]; Karimi et al. [22]; Mohammad et al. [23]; Negrut et al. [24]; Peng et al. [25]; Ranjbar et al. [26]; Tabowei et al. [27]; Valciukiene et al. [28]; Vorstenbosch et al. [29]; Yu et al. [30]; Zwezerijnen-Jiwa et al. [31]

In the analysis, we also used a matrix heatmap to visualize the interrelationship among the included studies. Each cell indicates the number of primary studies shared between the two included studies. Figure 2 shows how each included study obtained its data from unique sources, emphasizing overall diversity among the included studies.

Synthesis of Findings

This section presents a comprehensive synthesis of results on the gut microbiota's role in CRC diagnosis and development. In 20 studies, researchers have found a significant correlation between the disease and a range of microbial taxa. Hussan et al. and Peng et al. have demonstrated that Fusobacterium is a critical marker in CRC patients, as confirmed by several other studies showing its abundance in CRC tissue and fecal samples [21,25].

There is a considerable amount of interest in oral microbiota, with studies like Negrut et al. suggesting the use of salivary *Fusobacterium nucleatum* DNA as a noninvasive diagnostic method; this is supported by Yu et al. and Eastmond et al., who showed variations of oral cavity microbiota in the CRC [18,24,30].

Several studies, such as Gethings-Behncke et al., Ranjbar et al., Tabowei et al., and Valciukiene et al., emphasized the role of *F. nucleatum* in CRC diagnosis and prognosis and its potential as a treatment target [20,26-28]. In contrast, some studies, like Fratila et al., discussed the possible role of Lactobacilli and Bifidobacteria in CRC control [19]. Additionally, Zwezerijnen-Jiwa et al. suggested that combining traditional detection tests with microbiome markers can lead to more effective CRC detection [31].

Heterogeneity and Publication Bias

This umbrella review shows some heterogeneity among the reviewed papers because of the complex relationship between the CRC and gut microbiome, where methodological differences are seen, with some studies focusing on fecal samples while others focus on tissue samples. In addition, this article may have a review selection bias, favoring recent and peer-reviewed studies despite our efforts to include a variety of studies, and with positive publication bias becoming a problem in this discipline, we employed a detailed search in well-known databases to mitigate these.

Discussion

This umbrella review has thoroughly analyzed the existing literature on the role of the microbiota in colorectal cancer. This section addresses the potential implications, challenges, limitations, and future directions seen in the included studies.

Implications of the Results

As we comprehensively presented in the synthesis of results, there is a noteworthy correlation between dysbiosis in the gut and oral microbiota and CRC development and progression. Microbial markers, specifically *F. nucleatum*, can be low-cost, noninvasive tools for early detection, monitoring recurrence, and treatment response. Furthermore, integrating these microbial markers with traditional screening methods, like fecal occult blood tests, can increase the sensitivity and specificity of CRC diagnostics.

While the findings of studies on the correlation between specific microbiomes and CRC are promising, it is essential to acknowledge some challenges and limitations. Firstly, the cause-and-effect relationship between the disease and microbiomes is yet to be determined. Secondly, it is impractical to make direct comparisons due to different study methodologies like sample types (tissue vs. fecal), lab analysis techniques, and population demographics. Lastly, most studies did not account for variables that may influence the result, such as diet, age, genetics, and lifestyle, which can impact the gut microbiome.

Future Directions

There is a critical need for the standardization of microbiota methodologies, as it would allow comparable findings across studies. Future research should consider setting up large-scale multicenter longitudinal studies involving different populations and locations to verify the specific biomarkers needed to develop a universal diagnostic tool. Furthermore, studies should investigate how the microbiota influences CRC pathogenesis, leading to new approaches to targeted prevention and treatment therapies.

Bias, Flaws, and Low Study Overlap

According to a CA of 7.99% and a corrected CA of 3.15%, the studies we included have a low level of redundancy among the primary studies they covered, suggesting that a broad range of unique primary studies increases our findings' reliability while reducing the risk of citation bias.

## Conclusions

This umbrella review extensively analyzes the literature on the role of the microbiota in colorectal cancer. It strongly links gut and oral microbiota changes with CRC development. Our review paper suggests that the microbial marker *F. nucleatum* can be used as a noninvasive, cost-effective tool to improve early detection and monitoring of CRC. However, the current challenges are the varying methodologies and cause-and-effect between gut microbiota and CRC, which must be better understood. So, future research should focus on conducting large-scale longitudinal studies with standardized methodologies and exploring the mechanisms of gut microbiota influence on CRC for targeted therapeutics.
